# On Mental Disorders, Considred in a Medical, Hydienic, and Medico-Legal Point of View

**Published:** 1838-10-01

**Authors:** 


DES MALADIES MENTALES CONSIDERÉES SOUS LES RAPPORTS MEDICAL, HYGIENIQUE, ET MEDICO-LÉGAL. Par *E. Esquirol*, Médecin en Chef de la Maison Royale des Aliénés de Charenton, &c. &c. &c. Deux Tomes, accompagnés de 27 Planches Gravées. Chez J. B. Baillière, Paris et Londres, 1838.

ON MENTAL DISORDERS, CONSIDERED IN A MEDICAL, HYGIENIC, AND MEDICO-LEGAL POINT OF VIEW. By *E. Esquirol*, &c. &c. &c. Two Volumes, 8vo.

THE great space which we have devoted to the analysis of the Medico-Chirurgical Transactions and to the Life of Jenner in the present number, prevents our doing more than introduce these volumes to the notice of our readers. In our next number we shall render our account of them complete.

It would be absurd descanting, at this time of day, on the importance of an acquaintance with the nature and treatment of insanity. It would be equally absurd to dwell upon the difficulty of the study. Unfortunately, that difficulty is too often illustrated by pregnant instances in our Courts of Law; and medical witnesses continually break down beneath the keen cross-examination of subtle advocates. But the cause of their so often cutting a sorry figure is not simply the obscurity inherent in mental disorders—much of it must be laid to an imperfect information with respect to what is positively known of them, and much to that illogical method of reasoning so prevalent with members of our profession. The practice of medicine being essentially a calculation of chances, a mind not fortified by a sound education, is too apt to lose every thing like exactness, and to become entangled in the maze of probabilities. There are few who will not own how much they have been perplexed at times in grappling with this class of maladies, and fewer perhaps who will not peruse with curiosity and interest the experience of a man whose life has been spent in their investigation.

Such a man is M. Esquirol. The work before us is the produce of the observations and reflections of forty years, observations and reflections made upon those unhappy persons, amongst whom he has literally lived. Acquaintance with their habits taught him lessons of humanity, which he was not slow in learning, and M. Esquirol was among the first to denounce and abandon that cruel treatment of lunatics, which rivetted the madness that it found, and made the maniac the beast that it supposed him.

The experience of M. Esquirol has been gleaned at the Salpetriere, in the Asylum of Charenton, and in a wide field of private practice. His facts and opinions have been partially published at various times, in the Dictionnaire des Sciences Medicales, and in the Journals. He has never before collected them together in a corrected and methodical form, and he supposes, not without foundation, that their publication will not be unacceptable. We are sure that it will not.

The first Chapter of the work, intituled—De la Folie,—contains the principal generalizations of our author, the other chapters entering on details. To the first chapter, then, we shall proceed, and present an account of a large portion of its contents.

The mad-house, as M. Esquirol is at the pains of telling us, is a microcosm, where we see, *in petto*, the actors and the parts that appear upon the great stage of the world. Emperors and gods, rebels and subjects, devotees and infidels are on either scene, and if we smile at the madman's follies, it is because our participation in the corresponding follies of the sane, blunts the sense of the ridiculous in *them*. We may readily conceive some higher beings filled with pity and contempt, at the eagerness with which we pursue our fleeting objects of ambition.

But a philosophical glance at madness must not lead to so barren a conclusion. It should teach us to respect the delusions we observe, and to consider that they are founded on data as satisfactory to the insane, as the premises of our most demonstrative truths appear to be to ourselves. To contradict the delusion is, to the madman, what the obstinate contravention of the proposition is to us. The nature of the error ought to be investigated, the character of the lunatic should be considered. One we must encourage —another we must check—this person we engage by kindness—that we subdue by resolution—all we lead by hope. Such discrimination is the leading principle of modern treatment—coercion, chains and straw, were the alpha and omega of the ancient.

### I. Symptoms of Insanity.

M. Esquirol defines insanity as a cerebral affection, usually chronic, unattended with fever, and characterised by disordered conditions of sensibility, intelligence, or volition. But occasionally insanity runs a rapid course, and febrile symptoms are present.

The sensations of the insane are not in perfect correspondence with external objects. They will not read because the letters are distorted—they do not recognise their friends—are tormented with smells unappreciable by others. A lady, for example, in the last stage of phthisis was thus affected by the odour of charcoal. She imagined that a design was formed to asphyxiate her. She quitted her apartments, and changed her residence several times in a month. Phthisis destroyed her, retaining this illusion to the last.

These false sensations may affect one sense, often two, more rarely three, occasionally four, and even all. Alterations of hearing and of sight are, on the whole, most frequent. Many insane persons hear voices addressing them, and prompting them to all kinds of acts.

Another characteristic of insanity is the multiplicity of sensations and abundance of ideas, which occur without order, object, or stability. But we witness at other times, and very frequently, the reverse of this: the madman has one dominant idea, from which he cannot be diverted. This is monomania.

The proper associations of sensation and ideas undergo strange interruptions in insanity. The town of Die is commanded by a rock, named the V. A young man conceived the idea that by adding this letter to Die, he would make it Dieu (God), so he venerated all the inhabitants of Die as gods. But this Polytheism, after a time, not suiting him, he concentrated the Godhead in his father, as the most respectable person in the place.

In some insane persons, sensations are imperfect, impressions insufficiently felt, and the memory is faithless. These persons only recollect things long past, and they cannot fix their attention on existing objects.

In some cases, the madman seems no longer able to control his actions. An irresistible impulse forces him to acts that himself condemns. But he struggles against it in vain. The madman, too, as Locke observes, resembles those who reason correctly upon false premises. Thus a man murders his children, but he does so, because he thinks that, if they live, they will be damned.

The passions of madmen are impetuous, more especially in mania and in monomania. Many of the forms of madness are the natural passions carried to excess, and we pass, by gradual transitions, from the state of the most perfect calm to furious mania on the one hand, or to the deepest melancholy on the other.

The insane, although previously irreproachable, frequently commit the most disgraceful acts—indulge in gross obscenity, or perpetrate actual thefts. Their pusillanimity is remarkable. They are timid, suspicious, reserved. This trait is most conspicuous among those of least intelligence. M. Esquirol justly remarks that men of cultivated minds are the most candid and the least distrustful. But although the insane are filled with anxiety for the present moment, they are perfectly regardless of the future. And thus the distinctive mark of high civilization, a provident care for the morrow, would appear to be the very converse of madness. The

Carpe diem—
Nimium ne credula postero, however poetic, is the madman's motto.

The insane conceive an aversion for those who should be dear to them, and injure or fly from them. This would seem to be a consequence of their suspicions. If an apparent exception to this rule exists—if the madman appears to retain an affection for his relative or friend, he is deaf to his advice, his prayers, in whatever relates to his delusion. M. Esquirol insists upon this moral alienation, as an essential characteristic of the mental. There may be no apparent incoherence, but if the moral feelings are perverted, this is itself a sign of madness approaching or returning. The observation is, no doubt, a just one, when rightly understood, and properly weighed. But it is not to be supposed that every rascal is insane in the ordinary acceptation of the term, and can claim the indemnity of madness.

M. Esquirol makes a few observations on the physical states of the insane. But we see nothing in them to detain us. He affirms, in opposition to the popular belief, that there is usually something physically wrong about them.

When the insane are cured, they perfectly remember the sensations, true or false, that they have experienced, their reasonings, their resolves, even in detail. Jean Jacques said that "the state of reflection is unnatural, and the man who meditates a depraved animal." This rhodomontade is exactly the reverse of truth. But reasoning implies effort and attention; and it is the latter which is deficient in the insane. Imbeciles and idiots appear totally incapacitated for it. M. Esquirol has never succeeded in taking a plaister of Paris cast of the head of an imbecile. He never can keep his eyes closed long enough, however desirous he may be of having the cast taken of himself.

M. Esquirol conceives that there are good grounds for the quintuple division of insanity, in reference to its mental characters, into:—

1. Lypomania, (the melancholia of the ancients,) delusion in reference to one object, or to a few objects, with predominance of melancholy sentiments.

2. Monomania, delusion on a single object, or a few, with predominance of excited and of lively sentiments.

3. Mania, delusions of all sorts with excitement.

4. Dementia, where the reasoning faculty goes wrong, because its organs have lost their requisite energy and force.

5. Imbecilitas, in which the intellectual organs have never been capable of reasoning rightly.

Mental alienation may successively affect these different forms it is true, yet still, while they are preserved, they are distinct, and sufficiently indicative, in M. Esquirol's opinion, of appreciable mental conditions.

It has been a matter of inquiry, to ascertain the respective numbers of those affected with each of the preceding forms of insanity. M. Esquirol is of opinion that monomania is more frequent than mania—dementia, and imbecilitas, especially the latter, more rare; the latter, however, is endemic in some mountainous countries. See, for example, the Cretins of Switzerland.

### II. Causes of Insanity.

We shall not enter on their particular consideration, but we shall merely select a few particulars for notice.

A. *Climate.* Temperate climates, subject to great atmospheric variations, especially to alternations of cold and damp, and vice versâ, are those in which insanity is most prevalent. In some countries, insanity is endemic.

B. *Season.*—Unwonted heat and cold produce insanity. Many of the French soldiers went mad in Spain, and the same thing occurred in the retreat from Russia. But, in the latter case at all events, if not also in the former, moral causes must have operated. From a Table formed and published by our author, of the number of admissions into the Salpetriere during nine years, it appears:—1, that they were most numerous during the months of May, June, July, and August; 2, that the proportion decreased in September and December, to attain its minimum in February and March.

The seasons exercise an influence on insanity when actually developed. Some persons are depressed or excited during the Summer, and the reverse in the Winter. If insanity breaks out in the Spring or Summer, it has an acute form; if not quickly cured, they are decided in the Winter. The manias and monomanias of Autumn are decided in Spring. Summer is most favourable for the cure of dementia. Cures which take place during the warm season, though the most rare are the most durable. Relapses are most to be apprehended at the season of the year when the attack first happened, and they are most frequent in Spring and Summer. In some instances of intermittent madness, relapses occur regularly at the same seasons, even after intervals of several years.

M. Esquirol examines the question whether the moon exerts any influence on the insane. He cannot satisfy himself that it does so. If some are excited when the moon is at the full, it is, he thinks, on account of her light.

C. *Age.*—Though infancy is not the age for insanity, save and except idiotcy, yet Franck saw, in 1802, in St. Luke's Hospital, a child who has been insane from two years old. M. Esquirol has had the management of insanity in a child of eight years of age, in one of nine, and in one of eleven. On the whole, imbecility is the form which invades infancy: mania and monomania attack youth: lypomania or melancholia middle age: and dementia old age. From data which he slightly specifies, M. Esquirol concludes, that the age most prone to insanity in men, is that betweem 30 and 40 years—in women, between 50 and 60; and that the period of life least prone to madness is, in both sexes, infancy, youth, and advanced age. The rich are attacked in greater proportion than the poor at the age of 29 to 30.

D. *Sex.*—In Italy and Greece, women are less liable to insanity than men. In the North of France, more women are mad than men. In England, the equality between the sexes is greater. On this, as on several other occasions, M. Esquirol draws a melancholy picture of the moral state and position of women in France, a picture confirmed by the observation of all foreigners, and strikingly illustrated in her drama and her vicious modern novels. Society, in that country, is an anomalous state. The sanction of the Catholic religion has lost its hold upon the mind, and no corresponding one has been developed.

After examining several numerical data, M. Esquirol adds, that, when large numbers become the subject of calculation, the difference between the sexes, in reference to liability to insanity, is less than is commonly supposed —that this difference has a close approximate relation to the relative numbers of the sexes in the population—that the same ratio does not hold in all countries—and that, in France, more women are insane than in England.

Of those affected, a larger proportion of men are cured than women, and the former are less liable to relapses.

E. *Temperament.*—On this head, we see little to notice. We pass to—

F. *Professions and Modes of Life.*—Dryden observed, that "great wits to madness nearly are allied." M. Esquirol combats the position. He contends that persons of a wild and speculative turn of mind, and persons whose thoughts are constantly directed to one object are prone indeed to insanity. But those of the highest intellect have preserved it to a late period of existence. If painters, poets, musicians, &c., have evinced a tendency to madness, it is because their lives are, from circumstances, irregular.

The dominant idea of every age has had its influence in producing or in giving a colour to insanity. Thus at the dawn of Christianity religious melancholia prevailed—the age of chivalry gave birth to erotic melancholia—the wars of the reformation set afloat religious melancholia again—the modern aspirations for liberty and reform have turned many heads in France.

M. Esquirol lays it down as a sort of axiom that the frequency of insanity is always in the ratio of the dependence on social vicissitudes to which their professions expose men. Thus the palace and the bureau are severely visited, and courtiers, merchants, soldiers, suffer much from disease. A sedentary mode of life predisposes to it, and so does the sudden change from an active to an inactive one.

Occupations which expose those who pursue them to the vapor of charcoal, the heat of the sun, the metallic oxydes, &c. seem to give rise to insanity. Drunkenness, excessive venery, and particularly masturbation, do the same. The manners of a people and their morals, exercise a most potent influence. M. Esquirol dwells on the immorality of his country, and deplores its effects in the increase of insanity. The conscription laws, at each period of their operation, produced a sort of burst of insanity in France, either among the conscripts or their relatives. M. Esquirol examines the question, whether the numbers of the insane have materially increased since the revolution? He arrives at no satisfactory conclusion. The apparent numbers have greatly augmented, but that may be in a great degree explained by the multiplication of Asyla, and the greater care with which the patients are treated—inducements to all classes to send their mad relatives or friends.

G. *The Passions.*—M. Esquirol remarks that the most frequent moral causes of insanity are pride, terror, ambition, reverses of fortune, and domestic grief. The latter is a cause of extensive operation. M. Esquirol alludes to the idea that excessive joy gives rise to madness. He disputes it, and thinks that when "a man's head is turned with joy," some other subsidiary cause contributes to occasion the effect. In France, religious enthusiasm has ceased to make men mad—love, become a trade, no longer sends its votaries to the asylum. So, at least, says M. Esquirol, and he deplores the change which has come over his "unhappy country."

Moral causes are much more powerful in their operation than physical, particularly in the upper classes. Physical causes act more on women than on men, a circumstance which their menses, &c. explain.

H. *Physical Causes.*—Of these hereditary disposition is most powerful, especially amongst the wealthier portions of the people. It has made fools of many of the great families of France, who almost all intermarry. Insanity seems more transmissible by the mother than the father. Hereditary madness often appears in the parent and the child about the same age. A mother became insane, at the age of twenty-five, after an accouchement. Her daughter went mad at the same age, after confinement also. The children of insane persons frequently display peculiarities, which should forewarn those who are concerned in educating them. Such children should receive the physical and moral training which is calculated to prevent as much as possible all cerebral excitement.

Excitement of the mother, before or during gestation seems to give rise to a disposition to insanity in the offspring. M. Esquirol mentions several instances of this sort.

Falls or blows upon the head induce a disposition to mania.

Masturbation is both a prolific cause and an effect of it; continence a very rare one. Menstruation is a fruitful source of mania, especially the first menstruation, and the menstrual period is always attended with an aggravation of the malady. Suppression of the leucorrhœal discharge is a more frequent cause than is supposed. Pregnancy seems occasionally to give rise to insanity, but this is much more frequent after accouchement and during lactation. Dentition, producing convulsions, predisposes to insanity; the suppression of perspiration is a potent cause of it.

Fevers of a bad character leave after them a chronic delirium, which must not be confounded with insanity. But any cerebral affection may lay the foundation of that complaint. The presence of many substances in the primæ viæ, may give rise to it also, and chronic diseases, either by their suppression or metastasis, epilepsy, hysteria, apoplexy, the use of medicines which affect the nervous system, such as mercury, may produce the same effect.

We have run over M. Esquirol's account of the symptoms and the causes of insanity. Its course and treatment may be deferred until our next number, when we shall present an account of some, if not of all the forms which this deplorable infirmity assumes.

